# The Three-Dimensional Morphology of Femoral Medullary Cavity in the Developmental Dysplasia of the Hip

**DOI:** 10.3389/fbioe.2021.684832

**Published:** 2021-06-24

**Authors:** Min Zhang, Bo-Lun Liu, Xin-Zheng Qi, Qing-Qing Yang, Jing-Yang Sun, Qing-Yuan Zheng, Guo-Qiang Zhang, Cheng-Kung Cheng

**Affiliations:** ^1^Beijing Advanced Innovation Centre for Biomedical Engineering, School of Biological Science and Medical Engineering, Beihang University, Beijing, China; ^2^Department of Orthopedics, Chinese People’s Liberation Army General Hospital, Beijing, China; ^3^School of Biomedical Engineering, Shanghai Jiao Tong University, Shanghai, China

**Keywords:** DDH, hip morphology, femoral medullary cavity, femoral medullary torsion angle, femoral medullary roundness index, deformity of the femur

## Abstract

**Objective:**

This study aimed to assess the morphology of the femoral medullary canal in subjects with developmental dysplasia of the hip (DDH) with the intent of improving the design of femoral stems in total hip arthroplasty.

**Methods:**

Computed tomography images of 56 DDH hips, which were classified into Crowe I to Crowe IV, and 30 normal hips were collected and used to reconstruct three-dimensional morphology of the femoral medullary cavity. Images of twenty-one cross sections were taken from 20 mm above the apex of the lesser trochanter to the isthmus. The morphology of femoral cavity was evaluated on each cross section for the longest canal diameter, the femoral medullary torsion angle (FMTA), and the femoral medullary roundness index (FMRI).

**Results:**

The Crowe IV group displayed the narrowest medullary canal in the region superior to the end of the lesser trochanter, but then gradually aligned with the medullary diameter of the other groups down to the isthmus. The FMTA along the femoral cavity increased with the severity of DDH, but the rate of variation of FMTA along the femoral canal was consistent in the DDH groups. The DDH hips generally showed a larger FMRI than the normal hips, indicating more elliptical shapes.

**Conclusion:**

A femoral stem with a cone shape in the proximal femur and a cylindrical shape for the remainder down to the isthmus may benefit the subjects with severe DDH. This design could protect bone, recover excessive femoral anteversion and facilitate the implantation in the narrow medullary canal.

## Introduction

Developmental dysplasia of the hip (DDH), which is characterized by a shallow acetabulum, shortened femoral neck, excessive femoral anteversion and narrow femoral medullary cavity (Nakahara et al., 2014), may result in a range of complications to the hip joint such as dislocation, focal necrosis, and discrepancies in leg length (Naci and Kadri, 2011). Modular hip system and straight cone femoral stem are usually recommended for the treatment of DDH, due to the free setting of anteversion (Zhen et al., 2017; Wang, 2019; Gholson et al., 2020), and have shown good postoperative results (Gholson et al., 2020). However, difficult operative implantation of femoral stem in the patients with highly dislocated DDH hips (Liu et al., 2016) and intraoperative femoral fracture (Zang et al., 2019) were reported in previous clinical studies. Clinical experience suggests that the anatomic abnormalities inherent in the dysplastic femur increase with the degree of subluxation of the hip (Crowe et al., 1979; Sugano et al., 1998), thus the abnormal morphology of femoral medullary cavity in the DDH hips possibly limited the effective femoral stem implantation. Changes in the anatomy of the proximal femur associated with DDH can make implantation of a femoral stem challenging in cases where total hip arthroplasty is required (Yang and Cui, 2012). Good conformity between the femoral stem and the proximal femoral medullary cavity is required for stimulating bone ingrowth and improving initial stability and long-term fixation (Hayashi et al., 2015). However, few publications have reported on the detailed morphology of the femoral canal in subjects with DDH. In the previous study (Noble et al., 2003), CT scans were used to measure the medial-lateral width and anterior-posterior width of the proximal medullary canal in DDH patients. However, excessive femoral anteversion in DDH hips (Nakahara et al., 2014) is known to cause a twist in the femoral canal, and the medial-lateral width and anterior-posterior width may vary along the femoral medullary cavity. Liu et al. (2016) measured three cross sections of different regions of the femoral canal in DDH subjects, but the morphology variation in the whole proximal medullary cavity, where the femoral stem was fit, was still unknown and may limit the improvement of femoral stem designs.

The purpose of this study was to assess the morphology of the femoral medullary canal in the subjects with DDH with the intent of offering guidance for suitable femoral stem designs for total hip arthroplasty. The three-dimensional (3D) morphology of the femoral medullary cavity was reconstructed from computed tomography (CT) images of the femur. The femoral medullary cavity in DDH hips was classified according to the Crowe classification method (Crowe et al., 1979). The morphological characteristics of the femoral medullary canal in subjects with DDH were assessed regarding the size, torsion, and shape.

## Materials and Methods

### Subjects

The subject pool consisted of 56 adult patients (56 hips) who were diagnosed as DDH according to a comprehensive assessment with the lateral center edge angle [the lateral center edge angle <20° (Zhang et al., 2020)], acetabular angle [the acetabular angle >47° (Fujii et al., 2012)] and qualitative subluxation estimation, and were scheduled for total hip arthroplasty in our clinic from January 2019 to December 2019. The pool also included 30 healthy subjects with no signs of hip disease who acted as the control group (Table 1). Exclusion criteria were the presence of DDH with femoral fracture, bone defects, or bone tumors. All study protocols were approved by our institutional review board. Informed consent was obtained from all participants. Moreover, all the participants have consented to the publication of their data. Using the classification method detailed by Crowe et al. (1979), the final subject pool consisted of 26 subjects classified as Crowe I (less than 50% subluxation), nine as Crowe II (50 to 75% subluxation), seven as Crowe III (75 to 100% subluxation) and 14 as Crowe IV (more than 100% subluxation) (Table 1).

**TABLE 1:** Details of subject groups.

Group	Sample size	Gender		Affected side		Age (years)	Height (cm)	Weight (kg)
		Male	Female	Left	Right			
Control Group	30	21	9	10	20	30 (20 to 60)	172 (157 to 189)	70 (60 to 95)
DDH Group	56	3	53	32	24	46 (21 to 64)	159 (143 to 175)	62 (45 to 86)
DDH I	26	2	24	13	13	51 (26 to 63)	161 (153 to 175)	65 (55 to 86)
DDH II	9	1	8	5	4	44 (27 to 61)	162 (147 to 175)	59 (48 to 80)
DDH III	7	0	5	3	4	45 (31 to 64)	156 (143 to 161)	58 (45 to 75)
DDH IV	14	0	14	11	3	42 (21 to 64)	156 (143 to 163)	59 (45 to 80)

### Three-Dimensional Reconstruction of CT Images

The hips of all subjects (DDH and control groups) were scanned in a supine position with the lower limbs in neutral rotation using a multislice CT scanner (Discovery CT750 HD, GE Healthcare, United States) operating at 120 kV and 100 mA. All subjects were scanned from the superior margin of the ilium to one mm below the femoral condyle with a slice thickness of one mm. This region was chosen because of its importance in securing the femoral stem after a total hip replacement (Hayashi et al., 2015).

**FIGURE 1:**
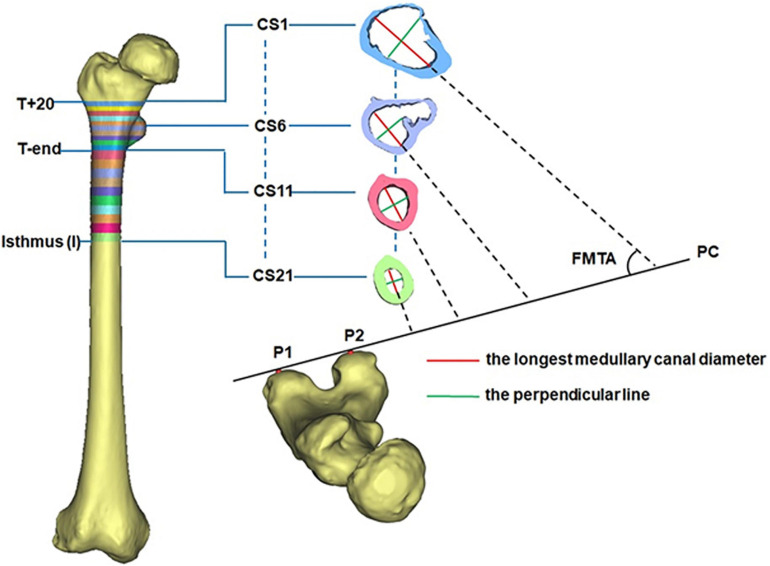
The morphological measurement of the femoral medullary cavity.

All CT images were recorded in DICOM format (512 × 512 pixels) and then imported into Mimics for 3D reconstruction (Mimics 17.0, Materialize, Leuven, Belgium). In Mimics, the femur was isolated from surrounding bone and soft tissues and then a 3D model of the femur was automatically reconstructed based on the default optimal settings. A femoral model was created for each of the DDH subjects and control subjects. The region from 20 mm above the apex of the lesser trochanter (T+20) to the isthmus was chosen for measurement because the medullary cavity in this span is critical for securing the femoral stem in a hip prosthesis (Hayashi et al., 2015). Due to individual variations in height, the region between T+20 and the end of the lesser trochanter (T-end) was evenly divided into ten intervals using nine cross sections (CS2 to CS10 in Figure 1), with the span ranging between 3.6 and 4.8 mm. The remaining region of each femur (T-end to the isthmus) was evenly divided into ten spaces (ranging from 7.2 to 8.7 mm) by the cross sections CS12 to CS20 in Figure 1. The cross sections on the levels of T+20, T-end, and isthmus were, respectively, marked as CS1, CS11, and CS21 (Figure 1).

### Measuring the Morphology of the Femoral Medullary Cavity

The 3D morphology of the femoral medullary canal was represented by three parameters: the longest canal diameter, the femoral medullary torsion angle (FMTA), and the femoral medullary roundness index (FMRI).

To reduce the effect of the femoral torsion on the measurements, the longest canal diameter (red line in Figure 1) in each cross section was chosen to assess the size of the femoral cavity. The built-in Measure Distance function in Mimics 17.0 (Materialize, Leuven, Belgium) (accuracy: 0.01 mm) was used to record the canal diameter and each measurement was repeated three times and then averaged. The resulting diameter of each cross section (CS1-CS21) was averaged for all femurs in the same group (DDH and control groups). This averaged value was used to represent the longest medullary canal diameter in that group. The variation in the longest canal diameter from T+20 to T-end and from T-end to isthmus were, respectively, calculated for each femur to investigate the change in the size of the femoral cavity.

The femoral medullary torsion angle was used to assess the degree of torsion within the canal. It was determined by the angle between the longest canal diameter (red line) and the posterior condyle (PC) line (Figure 1; Gaffney et al., 2019), measured using the MB-ruler software (Markus Bader, Germany) with the accuracy of 0.01 degrees. The PC line was determined by the two most prominent points on the posterior condyle (P1 and P2 in Figure 1; Gaffney et al., 2019). The femoral medullary torsion angle in each cross section from CS1 to CS21 was measured three times and the average was used as the value for that cross section. The femoral medullary torsion angle on each cross section was averaged for all femurs in the same group (DDH and control groups). This averaged value was used to represent the femoral medullary torsion angle for the cross section in that group.

The average rate of variation of femoral medullary torsion angle along the femoral cavity from T+20 to T-end and from T-end to isthmus was also calculated. These values were used to investigate how the femoral medullary torsion angle varies along the canal. Due to differences in individual heights, the rate of variation of femoral medullary torsion angle (*V*_*a–b*_) along the femoral medullary canal was defined by Eq. 1. In this equation, a and b were the cross section numbers, *FMTA*_*a*_ and *FMTA*_*b*_ were, respectively, the femoral medullary torsion angle values for cross section a and b, *distance*_*a*−*b*_ was the distance between the cross section a and the cross section b, and was measured with the built-in Measure Distance function (accuracy: 0.01 mm) in Mimics 17.0. As with the femoral medullary torsion angle values above, the rate of variation of femoral medullary torsion angle along the canal from T+20 to T-end and from T-end to isthmus was, respectively, calculated by *V*_*1–11*_ and *V*_*11–21*_ using Eq. 1. The results for *V*_*i–j*_ for the same region were averaged for all femurs in the same group and the average was used to represent the value for that group. A statistical investigation on the rate of variation of femoral medullary torsion angle along the femoral canal for each research group was performed.

(1) $$V_{a-b}=\left|\frac{F\,M\,T\,A_{a}-F\,M\,T\,A_{b}}{d\,i\,s\,t\,a\,n\,c\,e_{a-b}}\right|$$

The femoral medullary roundness index (FMRI) was used to evaluate the shape of the femoral medullary canal. The femoral medullary roundness index was defined as the ratio of the longest diameter of the medullary cavity to the width of its perpendicular diameter (green line in Figure 1) passing through the midpoint of the longest diameter. Measurement of the femoral medullary roundness index was repeated three times for each cross section and averaged. The value of the femoral medullary roundness index for each cross-section was then averaged in each group and used to represent the shape of the medullary cavity in that group.

### Accuracy of Measurement

The whole measurement process was repeated independently by three operators. The intraclass correlation coefficient (ICC) for the three operators was 0.912 for the longest canal diameter, 0.803 for the femoral medullary torsion angle (FMTA), and 0.813 for the femoral medullary roundness index (FMRI), suggesting good reliability across all measures (Weir, 2005). A univariate analysis was used to assess the inter-group differences for each cross section in the Crowe I, Crowe II, Crowe III, Crowe IV DDH groups and the control group with regard to the three parameters representing the 3D morphology of the femoral medullary canal (the longest canal diameter, the femoral medullary torsion angle, and the femoral medullary roundness index). The continuous variables were expressed as a mean and range. Shapiro-Wilk test was used to perform the normality test. The inter-group differences in the Crowe I, Crowe II, Crowe III, Crowe IV DDH groups, and the control group were assessed by using a one-way ANOVA test for the parametric variables and a Kruskal-Wallis test for the non-parametric variables. *A priori* power analysis with a significance level of 0.05 (type-I error), the desired power of 80%, and the effect size of 0.5 indicating a medium difference (Sullivan and Feinn, 2012) was performed for ANOVA and Kruskal-Wallis tests to evaluate the sample size. IBM SPSS 22.0 (IBM Corp., New York, NY, United States) was used for all statistical analyses. A *p* value less than 0.05 was considered significant.

## Results

### The Longest Medullary Canal Diameter

**FIGURE 2:**
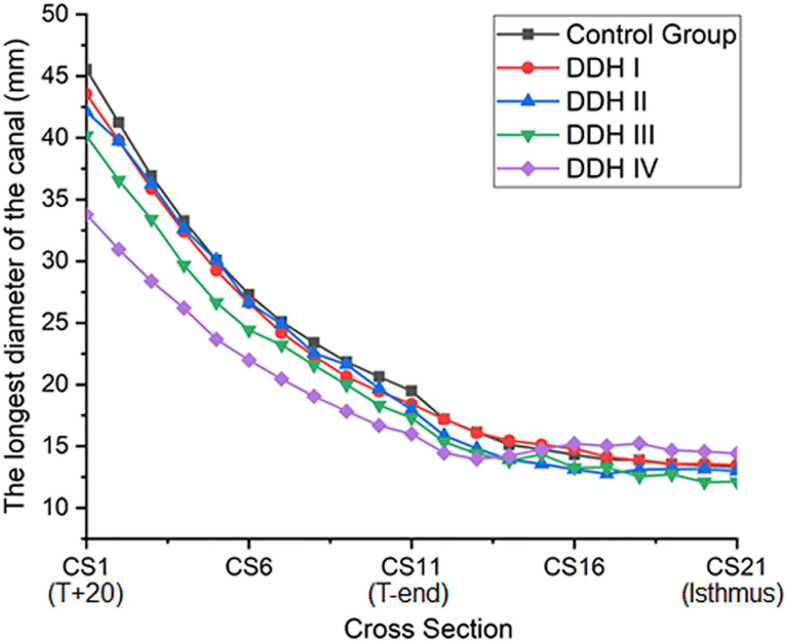
A plot showing the longest diameter of the femoral canal for all subjects.

The size of the medullary canal was determined by the longest canal diameter on each cross section from CS1 to CS21 (Figure 2). The results showed that there was a general decrease in canal diameter from the proximal to the distal femur (Figure 2). The variation in canal diameter from T+20 to T-end [Crowe I: 25.13 mm (16.66 to 34.45 mm); Crowe II: 24.14 mm (19.02 to 30.74 mm); Crowe III: 22.88 mm (10.56 to 28.19 mm); Crowe IV: 17.80 mm (7.61 to 26.43 mm)] was much larger than the variation from the T-end level to the isthmus level, with the values for the four Crowe types ranging between 4.67 to 5.17 mm (Table 2). Moreover, Crowe IV displayed an apparent shorter medullary canal diameter than those in control, Crowe I and Crowe II groups from T+20 to T-end (*p* < 0.001 for control and Crowe I groups, *p* = 0.021 for Crowe II group) and from T-end to isthmus (*p* < 0.001 for control group, *p* = 0.003 for Crowe I group, and *p* = 0.004 for Crowe II group) indicating a narrower canal. While there were no significant differences between Crowe III and Crowe IV in the variations of the longest diameter along the femoral canal (*p* = 0.157 for the variation between the T+20 and the T-end levels, and *p* = 0.097 for the variation between the T-end and the isthmus levels). Additionally, the medullary diameter in the subjects with severe DDH (Crowe III and Crowe IV groups) was not significantly different from those in the other groups on the isthmus level (*p* = 0.319 in Table 2).

**TABLE 2:** The longest diameter in all subject groups.

	Group					
	Control	DDH I	DDH II	DDH III	DDH IV	*p*-value across all the groups
T+20 (mm)	45.53 (36.48 to 54.38)	43.55 (32.24 to 53.05)	42.11 (37.42 to 45.97)	40.17 (21.02 to 46.99)	33.79 (24.41 to 44.43)	<0.001
T-end (mm)	19.49 (13.47 to 25.74)	18.42 (14.21 to 25.37)	17.97 (13.43 to 23.94)	17.29 (10.48 to 25.06)	15.99 (11.6 to 19.64)	0.014
Isthmus (mm)	13.32 (8.03 to 17.45)	13.47 (9.79 to 21.54)	13.00 (8.92 to 18.47)	12.12 (8.17 to 14.55)	11.32 (10.93 to 20.78)	0.319
Variation from T+20 to T-end (mm)	26.04 (16.72 to 33.75)	25.13 (16.66 to 34.45)	24.14 (19.02 to 30.74)	22.88 (10.56 to 28.19)	17.80 (7.61 to 26.43)	<0.001
Variation from T-end to isthmus (mm)	6.17 (1.67 to 12.41)	4.95 (1.65 to 9.28)	4.97 (4.00 to 6.32)	5.17 (2.31 to 10.51)	4.67 (0.75 to 6.49)	<0.001

### Femoral Medullary Torsion Angle (FMTA)

**FIGURE 3:**
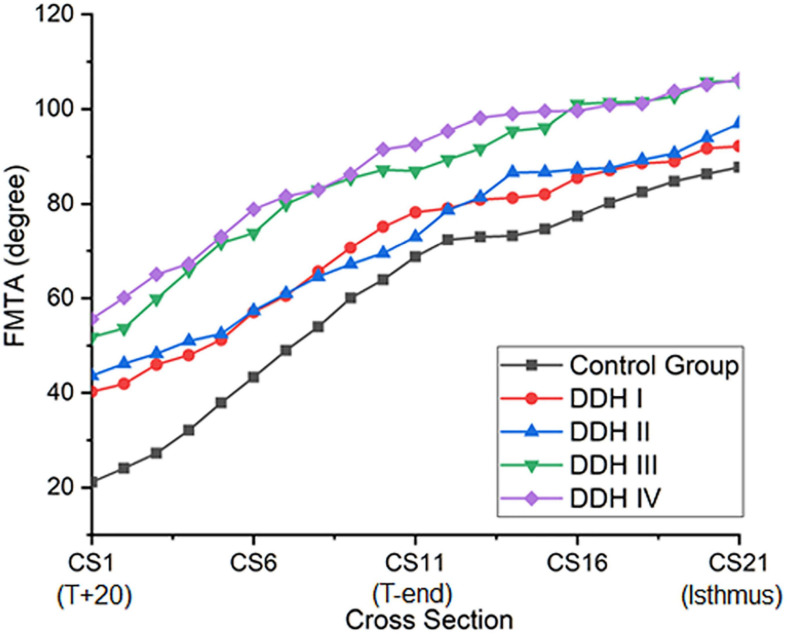
The plot of femoral medullary torsion angle (FMTA) for all subject groups.

All groups had a similar trend in femoral medullary torsion angle, with the angle increasing from proximal to distal (Figure 3). The average femoral medullary torsion angle at the T+20, T-end, and isthmus levels were shown in Table 3 and indicated that there were significant differences in all the groups (*p* < 0.001). A further study on the comparison between every two groups revealed that the FMTA in the healthy group was significantly lower than those in DDH groups (*p* < 0.001 for all the Crowe groups) on the T+20 level, as well as not significantly different from Crowe I (*p* = 0.711) and Crowe II (*p* = 0.387) and still lower than Crowe III (*p* = 0.011) and Crowe IV (*p* < 0.001) on the isthmus level.

**TABLE 3:** The femoral medullary torsion angle (FMTA) in all subject groups.

	Group					
	Control	DDH I	DDH II	DDH III	DDH IV	*p*-value across all the groups
T+20 (°)	21.15 (13.70 to 28.68)	40.27 (24.49 to 55.92)	43.65 (30.70 to 56.60)	51.79 (46.65 to 61.22)	55.62 (46.24 to 67.00)	<0.001
T-end (°)	68.75 (58.71 to 78.79)	78.23 (64.07 to 92.39)	73.01 (59.75 to 97.61)	86.94 (75.27 to 102.25)	92.59 (77.82 to 107.36)	<0.001
Isthmus (°)	87.75 (76.62 to 98.77)	92.21 (79.13 to 105.29)	96.99 (86.96 to 114.67)	105.91 (93.32 to 118.50)	106.25 (96.81 to 125.69)	<0.001

The rate of variation of femoral medullary torsion angle along the femoral cavity was significantly different in the research group (DDH and control groups) from T+20 to T-end (*p* = 0.002) and kept consistent from T-end to isthmus (*p* = 0.796) for all the subjects (Table 4). A further study on the DDH groups only indicated that the rate of variation of femoral medullary torsion angle along the canal didn’t vary significantly with the severity of DDH, with the *p* values from T+20 to T-end as well as that from T-end to isthmus being, respectively, 0.273 and 0.655.

**TABLE 4:** Detailed information on the variation of femoral medullary torsion angle (FMTA).

		Group					
		Control	DDH I	DDH II	DDH III	DDH IV	*p*-value across all the groups
Variation of FMTA (°)	T+20 to T-end	47.60 (45.01 to 50.11)	37.96 (36.47 to 39.58)	30.36 (28.05 to 41.01)	35.15 (28.62 to 51.03)	36.97 (31.58 to 40.36)	–
	T-end to isthmus	19.00 (17.91 to 19.91)	13.98 (12.90 to 15.06)	23.98 (17.07 to 27.21)	18.97 (16.25 to 20.05)	13.66 (11.65 to 18.33)	–
Distance (mm)	T+20 to T-end	38.08 (30.10 to 46.30)	36.93 (30.03 to 42.34)	35.40 (31.95 to 43.03)	35.53 (33.03 to 40.99)	37.89 (31.73 to 40.81)	–
	T-end to isthmus	85.13 (66.89 to 103.58)	78.49 (63.82 to 89.99)	82.60 (63.26 to 85.20)	83.17 (77.31 to 95.95)	80.52 (67.43 to 86.73)	–
The rate of variation in the FMTA over the femoral canal (°/mm)	T+20 to T-end	1.25 (1.08 to 1.50)	1.03 (0.93 to 1.21)	0.86 (0.80 to 0.91)	0.99 (0.87 to 1.22)	0.98 (0.91 to 1.03)	0.002
	T-end to isthmus	0.22 (0.19 to 0.25)	0.18 (0.16 to 0.21)	0.29 (0.27 to 0.32)	0.23 (0.20 to 0.24)	0.17 (0.15 to 0.21)	0.796

### Femoral Medullary Roundness Index (FMRI)

**FIGURE 4:**
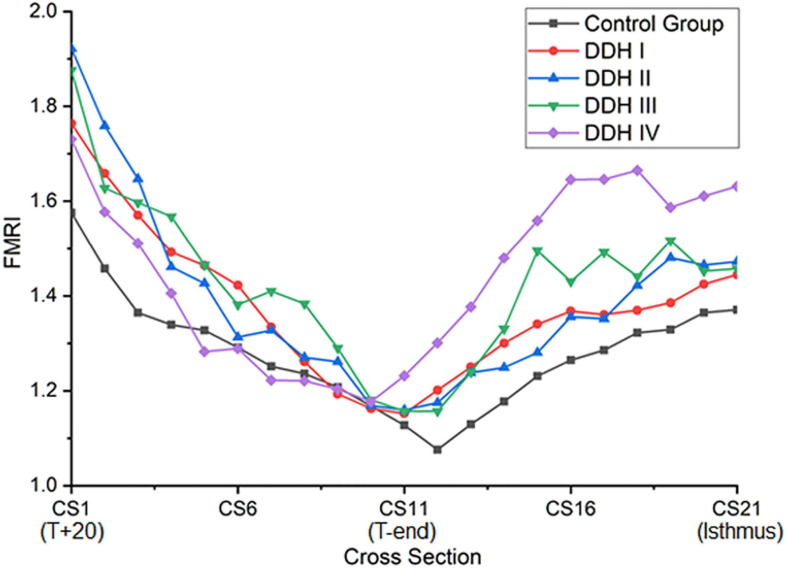
The plot of femoral medullary roundness index (FMRI) for all subject groups.

The graph in Figure 4 showed that all groups had a similar trend in the variation of femoral medullary roundness index over the length of the medullary cavity, with the femoral medullary roundness index decreasing from the T+20 level to the T-end level but then reverting as far as the isthmus. Also apparent was that the femoral medullary roundness index for the DDH group was generally larger than the control group from T+20 to T-end. These results signified that the shape of the femoral canal varied from an elliptical shape at T+20 to a more circular shape around T-end, and then reverted to be more elliptical again down to the isthmus. Moreover, the shape of the femoral canal for DDH subjects was generally more elliptical than the control subjects in the region of the lesser trochanter (Figure 4). The statistical analysis in Table 5 showed that the DDH groups had a generally larger femoral medullary roundness index than the control group, except for in the region around the end of the lesser trochanter (*p* = 0.689), and the femoral medullary roundness index for Crowe IV was significantly larger than the other groups at the isthmus level (*p* = 0.011).

**TABLE 5:** The femoral medullary roundness index (FMRI) in all subject groups.

	Group					
	Control	DDH I	DDH II	DDH III	DDH IV	*p*-value across all the groups
T+20	1.58 (1.33 to 1.83)	1.76 (1.45 to 2.07)	1.92 (1.47 to 2.37)	1.88 (1.30 to 2.46)	1.73 (1.15 to 2.31)	<0.001
T-end	1.13 (1.00 to 1.26)	1.15 (1.03 to 1.27)	1.16 (1.05 to 1.27)	1.16 (1.06 to 1.26)	1.19 (1.08 to 1.29)	0.689
Isthmus	1.37 (1.21 to 1.53)	1.44 (1.27 to 1.61)	1.47 (1.33 to 1.61)	1.46 (1.21 to 1.71)	1.63 (1.34 to 1.92)	0.011

## Discussion

This study aimed to evaluate the three-dimensional morphology of the femoral medullary canal in subjects with DDH with the intent to offer guidance for improved femoral stem design in total hip replacements. It was found that: (1) The Crowe IV group displayed the narrowest medullary canal in the region superior to the end of the lesser trochanter, but then gradually aligned with the medullary diameter of the other groups down to the isthmus. (2) The femoral medullary torsion angle along the femoral cavity increased with the severity of DDH, but the rate of variation of femoral medullary torsion angle along the femoral cavity was consistent among the DDH groups (Crowe I to Crowe IV). (3) The femoral medullary roundness index in the DDH groups was generally larger than the control group, except for around the end of the lesser trochanter.

In this study, the longest canal diameter was used to assess the size of the medullary canal. It was found that the Crowe IV hips showed the most severe narrowing of all research groups (DDH and control groups) (Figure 2). This result was in agreement with the studies by Sugano et al. (1998) and Noble et al. (2003), where the medullary cavity of DDH subjects was reported to be narrower than that of the control group, and the size of medullary canal reduced as the severity of the femoral deformity increased. The results of this current study also show that the medullary canal diameter in the DDH subjects reduced around the lesser trochanter (T+20 to T-end), but then varied only slightly from the T-end level to the isthmus (Table 2). The measurements of femoral medullary cavity suggest that a small stem with a large taper angle around the lesser trochanter (T+20 to T-end) and a small taper angle in the remaining region down as far as the isthmus (T-end to isthmus) may be suitable for Crowe IV hips. This design may reduce the high incidence of intraoperative femoral fracture caused by femoral stems with a consistent taper from the proximal femur to the isthmus (for example, the Wagner SL implant, Zimmer Inc., Warsaw, IN, United States) (Zang et al., 2019).

The results also showed that the femoral medullary torsion angle was larger in the DDH groups than in the control group and the value increased with the severity of DDH, which is in an agreement with Noble et al. (2003). The femoral medullary torsion angle values at the T+20, T-end, and Isthmus levels in our study (Table 3) were, respectively, 21.15° (13.70° to 28.68°), 68.75° (58.71° to 78.79°) and 87.75° (76.62° to 98.77°) for the control group and 55.62° (46.24° to 67.00°), 92.59° (77.82° to 107.36°), and 106.25° (96.81° to 125.69°) for the Crowe IV group. Similarly, the reported angles at these three levels in Noble’s study (Noble et al., 2003) were 30°, 59° and 86° in the control group and 58°, 77°, and 98° in the Crowe IV group. Additionally, it was found that the rotation angle from T+20 to the isthmus was the largest in the control group (66.60°), followed by Crowe I (51.94°), Crowe II (53.34°), Crowe III (54.12°), and Crowe IV (50.63°) (Table 4). The results are largely in agreement with those reported in the literature (Sugano et al., 1998), in which the femoral canal was reported to be twisted at 48° in normal hips, and then smaller angles for DDH groups (Crowe I: 36°, Crowe II and Crowe III: 42°, Crowe IV: 37°). The slight difference may be explained by the fact that different sections of the femur were chosen for analysis. In our study, the targeted range of the rotation was from 20 mm proximal to the apex of the lesser trochanter to the isthmus, while (Noble et al., 2003) considered from 20 mm distal to the center of the lesser trochanter to the canal isthmus. Other possible causes for the variation may be down to differences in the subject population, sex, as well as methods used for measuring the medullary canal. However, the discrepancies between the results of this study and those reported by Sugano et al. (1998) and Noble et al. (2003) were minor and the change in femoral medullary torsion angle in the DDH and control groups showed a similar trend between these studies. Considering the rate of variation of femoral medullary torsion angle along the femoral cavity, the results in this study indicated that the femoral cavity in the DDH groups twisted at a consistent rate of variation, and the value in the DDH groups was smaller than that in the control group in the proximal femoral region. The results indicated that the difference in femoral medullary torsion angle between the DDH and control medullary canals was possibly caused by the femoral anteversion, which refers to the rotation of the femoral neck around the diaphysis. A normal femoral anteversion is beneficial to restoring the normal biomechanics of the dysplastic joint (Noble et al., 2003; Wang, 2019), as well as improving the postoperative durable implant fixation and joint mobility (Dorr et al., 2009), thus a suitable femoral replacement design for patients with severe DDH may need to consider correcting the excessive femoral anteversion. This could be achieved using a modular stem with a rotatable sleeve (Liu et al., 2016), or a cone stem (Zhen et al., 2017). Alternatively, additive manufacturing is a developing technology that has shown promising results for achieving a close match to the individual anatomy of the femoral medullary cavity (Hua et al., 2010). Similar techniques could be used to create a customized uncemented femoral stem for patients with severe DDH to recover excessive femoral neck anteversion.

The shape of the femoral canal was assessed through the femoral medullary roundness index. It was found that the femoral medullary roundness index in all subjects decreased from the level of T+20 down to T-end, and then increased down to the isthmus. In the region around T-end, there was no significant difference in femoral medullary roundness index between all groups (control and DDH groups). The results also showed that the shape of the femoral canal varied from an elliptical shape at T+20 to a more circular shape around T-end, and then reverted to an ellipse down to the isthmus. In addition, the femoral roundness index for the DDH groups was generally larger than the healthy subjects, indicating a more elliptical shape in the DDH hips. Considering the twisted femoral medullary canal, the elliptical cross section shape of the femoral cavity indicated that a femoral stem with a circular cross section may be beneficial for protecting bone. Clinical studies on cone-shaped hip stems (Wagner cone prosthesis hip stem, Zimmer^®^, United States) have shown good recovery of excessive femoral anteversion, stable proximal fixation, and good survivorship for the patients with DDH (Zhen et al., 2017). Considering the size of the femoral canal reduced steeply only in the region superior to the end of the lesser trochanter and changed slightly in the remaining distal region down to the isthmus (Figure 2), a femoral stem with a cone shape in the lesser trochanter and a cylindrical shape down to the isthmus is recommended for patients with severe DDH (i.e., Crowe IV), which is characterized by a large femoral medullary rotational angle and narrow metaphyseal canal. This design may reduce interoperative bone fracture, offer good conformity between the femoral stem and the proximal femoral medullary cavity, as well as protect bone. The suitable design of femoral stem for DDH is not only beneficial to improve gait performance but helpful for trunk activities, due to the correlation between gait and paraspinal muscle activation (Miscusi et al., 2019).

A limitation of this study was that the number of the research subjects was limited, as well as the number and sex of subjects assigned to each of the DDH groups and the control group were not consistent. Nevertheless, the femoral medullary torsion angle results in this study were consistent with those reported by Sugano et al. (1998) and Noble et al. (2003) and confirm the reliability of the model used in this study. The second limitation was that the femoral stem design with a cone shape around the lesser trochanter and a cylinder shape for the remaining region down to the isthmus was only recommended based on the study on the morphology of femur in the subjects with DDH, a further comprehensive assessment for the proposed stem design could be performed in the future.

In conclusion, the subjects with severe DDH deformities displayed a narrow medullary canal in the region only superior to the end of the lesser trochanter, and then a slight variation in the canal size from the other groups for the remainder of the canal down to the isthmus. In addition, the severe DDH group had a larger twist and a more elliptical-shaped canal in comparison to the healthy subjects. The characteristics of morphology of femur with severe DDH deformities indicated that a femoral stem with a cone shape in the region only superior to the end of the lesser trochanter and a cylinder shape for the remaining region down to the isthmus may benefit the subjects with severe DDH, protecting bone, recovering excessive femoral anteversion and facilitating the implantation in the narrow medullary canal.

## Data Availability Statement

The raw data supporting the conclusions of this article will be made available by the authors, without undue reservation.

## Ethics Statement

Written informed consent was obtained from the individual(s) for the publication of any potentially identifiable images or data included in this article.

## Author Contributions

MZ, G-QZ, and C-KC: conceptualization. MZ, Q-QY, and C-KC: methodology. MZ, B-LL, X-ZQ, J-YS, and Q-YZ: formal analysis and investigation. MZ: writing – original draft preparation. B-LL, X-ZQ, Q-QY, J-YS, Q-YZ, G-QZ, and C-KC: writing, review, and editing. G-QZ and C-KC: supervision. All authors have agreed to be accountable for the content of the work.

## Conflict of Interest

The authors declare that the research was conducted in the absence of any commercial or financial relationships that could be construed as a potential conflict of interest.

## Abbreviations

CT: computed tomography
DDH: developmental dysplasia of the hip
FMRI: femoral medullary roundness index
FMTA: femoral medullary torsion angle.

## References

[B1] Crowe J. Mani V. Ranawat C. (1979). Total hip replacement in congenital dysplasia and dislocation of the hip. *J. Bone Joint Surg. Am.* 61 15–23. 10.1007/978-1-4471-5451-8_30 365863

[B2] Dorr L. D. Malik A. Dastane M. Wan Z. (2009). Combined anteversion technique for total hip arthroplasty. *Clin. Orthop. Relat. Res.* 467 119–127. 10.1007/s11999-008-0598-4 18979146 PMC2600986

[B3] Fujii M. Nakashima Y. Sato T. Akiyama M. Iwamoto Y. (2012). Acetabular tilt correlates with acetabular version and coverage in hip dysplasia. *Clin. Orthop. Relat. Res.* 470 2827–2835. 10.1007/s11999-012-2370-z 22544668 PMC3441999

[B4] Gaffney B. M. Hillen T. J. Nepple J. J. Clohisy J. C. Harris M. D. (2019). Statistical shape modeling of femur shape variability in female patients with hip dysplasia. *J. Orthop. Res.* 37 665–673. 10.1002/jor.24214 30656719 PMC6613213

[B5] Gholson J. J. Wallace S. S. Akram F. Gonzalez A. Kunze K. N. Levine B. R. (2020). Wagner cone midterm survivorship and outcomes. *J. Arthroplasty* 35 2155–2160. 10.1016/j.arth.2020.03.015 32279943

[B6] Hayashi S. Fujishiro T. Hashimoto S. Kanzaki N. Kuroda R. Kurosaka M. (2015). The contributing factors of tapered wedge stem alignment during mini-invasive total hip arthroplasty. *J. Orthop. Surg. Res.* 10:52. 10.1186/s13018-015-0192-x 25895960 PMC4410464

[B7] Hua J. Walker P. S. Muirhead-Allwood S. K. Engelhardt F. Bentley G. (2010). Custom uncemented revision stems based on a femoral classification. *Hip. Int.* 20 18–25. 10.1016/j.gaitpost.2009.09.011 20235071

[B8] Liu S. Zuo J. Li Z. Yang Y. Liu T. Xiao J. (2016). Study of three-dimensional morphology of the proximal femur in developmental adult dysplasia of the hip suggests that the on-shelf modular prosthesis may not be an ideal choice for patients with Crowe type IV hips. *Int. Orthop.* 41 707–713. 10.1007/s00264-016-3248-6 27416867

[B9] Miscusi M. Serrao M. Conte C. Ippolito G. Marinozzi F. Bini F. (2019). Spatial and temporal characteristics of the spine muscles activation during walking in patients with lumbar instability due to degenerative lumbar disk disease: evaluation in pre-surgical setting. *Hum. Mov. Sci.* 66 371–382. 10.1016/j.humov.2019.05.013 31153034

[B10] Naci E. Kadri Y. (2011). A study on the complications of surgical treatment for bilateral developmental dysplasia of the hip and a comparison of two osteotomy techniques. *Eurasian J. Med.* 43 162–168. 10.5152/eajm.2011.38 25610185 PMC4261396

[B11] Nakahara I. Takao M. Sakai T. Miki H. Nishii T. Sugano N. (2014). Three-dimensional morphology and bony range of movement in hip joints in patients with hip dysplasia. *Bone Joint J.* 96 580–589. 10.1302/0301-620X.96B5.32503 24788490

[B12] Noble P. C. Kamaric E. Sugano N. Matsubara M. Harada Y. Ohzono K. (2003). Three-dimensional shape of the dysplastic femur: implications for THR. *Clin. Orthop. Relat. Res.* 1 27–40. 14646700

[B13] Sugano N. Noble P. C. Kamaric E. Salama J. K. Ochi T. Tullos H. S. (1998). The morphology of the femur in developmental dysplasia of the hip. *J. Bone Joint Surg. Br.* 80 711–719. 10.1302/0301-620x.80b4.0800711 9699842

[B14] Sullivan G. M. Feinn R. (2012). Using effect size—or why the P value is not enough. *J. Grad. Med. Educ.* 4:279. 10.4300/JGME-D-12-00156.1 PMC3444174 23997866

[B15] Wang Y. (2019). Current concepts in developmental dysplasia of the hip and Total hip arthroplasty. *Arthroplasty* 1:2. 10.1186/s42836-019-0004-6 PMC8787940 35240757

[B16] Weir J. P. (2005). Quantifying test-retest reliability using the intraclass correlation coefficient and the SEM. *J. Strength Cond. Res.* 19 231–240. 15705040 10.1519/15184.1

[B17] Yang S. Cui Q. (2012). Total hip arthroplasty in developmental dysplasia of the hip: review of anatomy, techniques and outcomes. *World J. Orthop.* 3 42–48. 10.5312/wjo.v3.i5.42 22655221 PMC3364316

[B18] Zang J. Uchiyama K. Moriya M. Fukushima K. Takahira N. Takaso M. (2019). Long-term outcomes of Wagner self-locking stem with bone allograft for Paprosky type II and III bone defects in revision total hip arthroplasty: a mean 15.7-year follow-up. *J. Orthop. Surg.* 27:2309499019854156. 10.1177/2309499019854156 31181993

[B19] Zhang D. Pan X. Zhang H. Luo D. Cheng H. Xiao K. (2020). The lateral center-edge angle as radiographic selection criteria for periacetabular osteotomy for developmental dysplasia of the hip in patients aged above 13 years. *BMC Musculoskelet. Disord.* 21:493. 10.1186/s12891-020-03515-8 32711501 PMC7382803

[B20] Zhen P. Liu J. Lu H. Chen H. Li X. Zhou S. (2017). Developmental hip dysplasia treated by total hip arthroplasty using a cementless Wagner cone stem in young adult patients with a small physique. *BMC Musculoskelet. Disord.* 18:192. 10.1186/s12891-017-1554-9 28506299 PMC5432993

